# Factors associated with sexually transmitted reinfections, number of sexual partners and condom use among previously infected young people

**DOI:** 10.1177/09564624251348693

**Published:** 2025-06-11

**Authors:** Isobel Landray, James R. Carpenter, Sima Berendes, Melissa J. Palmer, Caroline Free

**Affiliations:** 1 Department of Medical Statistics, Faculty of Epidemiology and Population Health, 4906 London School of Hygiene & Tropical Medicine, London, UK; 2 Department of Public Health, Environments and Society, Faculty of Public Health and Policy, 4906 London School of Hygiene & Tropical Medicine, London, UK

**Keywords:** Sexual behaviour, chlamydia, gonorrhoea, high-risk behaviour, condoms

## Abstract

**Background:**

Sexually transmitted infections (STIs) are prevalent in young people. Digital interventions promoting safer sexual behaviours are low-cost and scalable. We use data from a randomised controlled trial of one such potential intervention (safetxt) to investigate factors associated with STI reinfection and risky sexual behaviours.

**Methods:**

We use data from 6248 young people with STIs recruited from 92 UK sexual health clinics. Multivariable logistic regression models were developed with the outcomes: reinfection, condom use at last sex and number of sexual partners (≤1 or >1) at 1 year. A pre-specified variable selection process assessed effects of sociodemographic and sexual behaviour factors measured at trial baseline.

**Results:**

Factors associated with reinfection included sexuality, ethnicity, baseline diagnosis of gonorrhoea and chlamydia, index of multiple deprivation, whether the participant and/or the last new partner tested before sex. Risk factors for condom use at last sex and number of sexual partners included sexuality and education level. The multivariable models had good calibration but poor discrimination.

**Conclusions:**

In this large sample with good representation across social and ethnic groups, we identified patient characteristics associated with higher risk of reinfection. Improved understanding of factors associated with reinfections and higher-risk sexual behaviours can aid development of interventions.

**Trial Registration:**

ISRCTN64390461.

## Introduction

Sexually transmitted infections (STIs), in particular chlamydia and gonorrhoea, are a major global health concern in terms of their burden and consequent long-term adverse health effects. Risk factors associated with STI transmission include unprotected sex with multiple partners and history of STIs.^1^ STIs such as chlamydia and gonorrhoea are most common in young people aged 15–24 years.^2^ Among other factors such as underlying levels of infection within sexual networks, social and economic circumstances, policies and access to care, young people’s behaviours are drivers of their higher burden of STIs.^3^ These behaviours include inconsistent condom use and tendency to have more sexual partners. It is vital to understand the factors associated with these behaviours.

The risk of adverse health effects increases with more infections.^4^ Younger age and being a man-who-has-sex-with-men (MSM) are associated with a higher risk of reinfection.^5^ Several studies have highlighted structural causes for inequalities for STI infection that particularly impact socioeconomically deprived and some ethnic minority populations.^6,7^ However, inequalities in patterns of reinfection have been subject to less investigation.

In most STIs, infection does not result in strong, lasting protective immunity so reinfections are common, including following treatment.^8,9^ The rates of reinfection at 1 year following treatment are up to 30% for chlamydia and 12% for gonorrhoea.^5^ The risk of adverse health effects, such as pelvic inflammatory disease, increases with more infections.^4,10^

The safetxt Randomised Control Trial (RCT)^11,12^ recruited young people in the UK who had recently been diagnosed with chlamydia, gonorrhoea or non-specfic urethritis (NSU) to assess the effect of a novel text-messaging based intervention ('safetxt'). The primary outcome was the cumulative incidence of STI reinfection at 1 year, which had a high follow-up rate. While the intervention was not found to be of benefit for the primary outcome, qualitative feedback from participants was positive and attributed increased condom use, STI testing, and confidence in partner notification to their receipt of the intervention.^13^

The safetxt dataset forms a large prospective cohort of 16–24 years olds living in the UK and provides the opportunity to improve understanding of individuals’ risks of STI reinfection and factors associated with higher-risk sexual behaviours. A previously published secondary analysis of these data found that the odds of STI reinfection and condom use at 1 year varied according to participant age, sex, and sexuality.^14^ The objectives of this analysis are to further assess associations between other demographic variables and safer sex practices (e.g. testing) with: reinfection, condom use, and multiple sexual partners. Understanding the factors associated with higher risk of reinfection as well as the dynamics surrounding condom use and having multiple sexual partners, can inform future work to develop targeted interventions.

## Methods

### Data source

The safetxt RCT provides individual level data for 6248 trial participants recruited from 92 UK sexual health clinics.^11^ All participants were 16–24 years old and had a diagnosis of chlamydia, gonorrhoea, or NSU at baseline. A high proportion of participants had complete follow-up data for reinfection at 1 year: 74.8%.

### Safetxt intervention

Safetxt was a series of automated text messages sent over 1 year.^4,11,15^ Safetxt’s aim was to reduce STI reinfection by increasing partner notification, condom use and STI testing before sex with a new partner. The message content and schedule were tailored by sex/gender identity (female, male, non-binary), sexual orientation (sex/gender of previous sexual partners) and infection type at baseline.

### Measures

Full details of measures assessed are reported in Supplemental File 1.^4,11,15^ Here, the measures relevant to this analysis are outlined.

Twelve variables measured at trial baseline were assessed for possible associations with the outcomes of interest. Socio-demographic and sexual health behaviour variables measured at baseline were: age, sexuality group, ethnicity (self-defined according to UK census groupings), type of infection, education level (primary and secondary (age ≤16 years), secondary onwards (age ≥17 years), still in full time education), index of multiple deprivation (IMD,^16^ note that this is similar to the United States Social Vulnerability Index), condom use during last sexual encounter, condom use during first sexual encounter with last new partner, tested before sex with last new partner, partner tested before sex with last new partner and number of partners in past 12 months (0, 1, ≥2). Sexuality was grouped as specified in the trial Statistical Analysis Plan (SAP): MSM or men-who-have-sex-with-men-and-women (MSMW); men-who-have-sex-with-women-only (MSW); women-who-have-sex-with-men (WSM) or women-who-have-sex-with-men-and-women (WSMW); women-who-have-sex-with-women-only (WSW); all other groups (non-binary individuals and individuals that did not state their sexuality were grouped due to data sparsity).^12^ Any response of “unsure” in the self-reported sexual health variables were considered missing. The allocation arm (safetxt vs control) was considered a potential risk factor, as done by Pocock et al.^17^

The outcomes of interest were reinfection (primary trial outcome: cumulative incidence of chlamydia or gonorrhoea reinfection at 1 year), condom use at last sexual encounter and number of sexual partners (≤ or >1 partners) at 1 year. Reinfection was assessed using self-sampling kits that were posted to all respondents at 12 months.^4^ Additionally, data from STI tests completed during the 12 months follow-up period were obtained from clinical records. The assessment of these are described in more detail elsewhere.^4,11,15^

To aid in imputation models, scores were derived from variables collected at 4-week follow-up which measured: attitudes towards partner notification, self-efficacy in telling a partner about an infection, self-efficacy in negotiating condom use, correct condom use self-efficacy, and knowledge related to STIs (full details of scores are presented in Table S1 and Appendix 2 of RCT paper).^11^

### Statistical analysis

Analyses were conducted using Stata 18.5.

#### Model development

Three models are developed, one for each outcome of interest at 1 year:

(1) Reinfection
(2) Condom use at last sexual encounter
(3) Multiple sexual partners

#### Multiple imputation

To avoid failing to detect important predictors and estimates being biased due to missing data, multiply imputed (MI) datasets are used.^11,12^ One hundred MI datasets are formed using multivariate imputation by chained equations (MICE) (Suppl 2).

#### Model selection

For each outcome, the following model selection process is followed. Firstly, 12 logistic models were fitted individually with each risk factor to assess their unadjusted effects. If age was significant, a model with age-squared was fitted. Age was taken as the number of years older than 16. Any of the statistically significant factors in the unadjusted models were included in a backwards variable selection process along with their two-way interactions. Backwards variable selection ‘locks’ the main effects and assesses the significance of the interaction terms (cut-off *p* > .05). Then, the backwards variable selection is repeated only ‘locking’ main effects whose interaction terms were included to remove any main effects that have become non-significant.^17^

#### Model assessment

By treating performance measures as parameters, MI estimates are obtained by Rubin’s rules^18^ (Suppl 3).

Accuracy is estimated as a proportion. Calibration is tested using Hosmer-Lemeshow’s test. Discrimination is assessed using area under the receiver operating characteristic curve (AUC); AUC close to one indicates very good discrimination. AUC is also calculated using cross-validation (CV) with 10 folds for internal validation.

## Results

Suppl 4 summarises baseline socio-demographic and sexual-health related variables.^11^ Suppl 5 presents the primary trial outcome results of interest in this paper.^11^

### Reinfection

The following main effects were selected: allocation arm, age, sexuality group, ethnicity, type of infection at baseline, IMD, whether the participant tested before sex with last new partner and whether the participant’s most recent new partner tested before sex with their last new partner. The interactions between age and sexuality, allocation arm and ethnicity, and age and whether the participant’s most recent new partner tested before sex with their last new partner were included. This means that the effect of age on reinfection depended on the participant’s sexuality and whether the participant’s most recent new partner had tested before sex with their last new partner.

The following interpretations are conditional on the model’s other covariates (Table 1). The odds of reinfection for a MSM or MSMW individual whose most recent new partner did not test before sex with their last new partner increase by approximately 20% for each year older the individual is (OR 1.19, 95% CI: 1.06 to 1.33, *p* = .002). Respectively on the safetxt and control arms, the ethnicities associated with the highest odds of reinfection were ‘other’ background and Asian/Asian British. Black/Black British participants have higher odds of reinfection compared to white participants (*p* < .001). The odds of reinfection for someone with gonorrhoea and chlamydia are 1.76 times those of someone with chlamydia alone at baseline (95% CI: 1.32 to 2.35, *p* < .001). The odds of reinfection for someone in a “most deprived” area are 1.49 times those of someone in a “least deprived” area (95% CI: 1.16 to 1.92, *p* = .002).

**Table 1.** Results of prognostic models for reinfection at 1 year developed using multiply imputed datasets.

	Coefficient^†^ (95% CI)	*p*-value
Intercept/Reference*	−1.88 (−2.57, −1.19)	<0.001
**Safetxt allocation**	0.18 (0.00, 0.35)	0.045
**Age (years)**	0.17 (0.06, 0.28)	0.002
**Sexuality group**		*0.087*
MSW only	−0.05 (−0.79, 0.70)	0.904
WSM or WSMW	0.50 (−0.15, 1.15)	0.133
WSW only	−1.13 (−4.56, 2.29)	0.517
All other groups	−1.09 (−5.35, 3.17)	0.615
**Age (years)-sexuality interaction**		*<0.001*
MSW only	−0.21 (−0.33, −0.07)	0.002
WSM or WSMW	−0.29 (−0.40, −0.18)	<0.001
WSW only	−0.28 (−1.14, 0.57)	0.519
All other groups	−0.07 (−0.99, 0.85)	0.883
**Ethnicity**		*<0.001*
Black/Black British	0.74 (0.44, 1.04)	<0.001
Asian/Asian British	1.12 (0.58, 1.65)	<0.001
Mixed background	0.37 (−0.02, 0.76)	0.062
Other background	0.43 (−0.44, 1.31)	0.333
**Safetxt-ethnicity interaction**		*0.049*
Black/Black British	−0.15 (−0.55, 0.26)	0.479
Asian/Asian British	−1.18 (−2.05, −0.33)	0.007
Mixed background	0.27 (−0.30, 0.84)	0.355
Other background	0.33 (−0.81, 1.46)	0.574
**Type of infection**		*<0.001*
Gonorrhoea	−0.07 (−0.33, 0.18)	0.573
Gonorrhoea and chlamydia	0.56 (0.28, 0.85)	<0.001
Gonorrhoea or NSU	0.07 (−0.66, 0.81)	0.845
NSU	−0.57 (−1.08, −0.05)	0.032
Don’t know	−0.38 (−0.93, 0.17)	0.174
**IMD**		*<0.001*
2	−0.06 (−0.33, 0.21)	0.660
3	0.05 (−0.21, 0.31)	0.692
4	0.24 (−0.01, 0.49)	0.055
5 – most deprived	0.40 (0.15, 0.65)	0.002
**Tested before sex with last new partner**	0.17 (0.01, 0.33)	0.036
**Most recent new partner tested before sex with their last new partner**	0.36 (−0.07, 0.79)	0.101
**Age (years)-most recent new partner tested before sex interaction**	−0.08 (−0.17, 0.01)	0.096

All predictor variables are measured at baseline. Joint *p*-values are in *italics*.

CI = confidence interval; IMD = index of multiple deprivation; MSW = men who have sex with women; WSM = women who have sex with men; WSMW = women who have sex with men and women; WSW = women who have sex with women; NSU = non-specific urethritis.

†Coefficients are log-odds ratios. *Reference levels are as follows: control arm, 16 years old, men who have sex with men or men who have sex with men and women, white, chlamydia infection at baseline, IMD of 1 (least deprived), did not test before sex with last new partner and their partner did not test before sex with last new partner.

Table 2 shows the model assessment measures for all the models. The model’s accuracy was good: 0.79. The AUC and CV AUC were poor: 0.67 and 0.64, respectively. The goodness-of-fit test concluded there was no evidence of poor fit or calibration and good performance across the risk strata ( $F_{3,1809}$ = 0.30, *p* = .83).

**Table 2.** Model assessment measures for prognostic models for reinfection, condom use at last sexual encounter and number of sexual partners at 1 year developed using multiply imputed datasets.

	Reinfection	Condom use at last sexual encounter	Number of sexual partners
**Accuracy**	0.79	0.68	0.63
**AUC**	0.67	0.62	0.66
**AUC (10-fold cross validation)**	0.64	0.61	0.65
**Goodness of fit test**	$F_{3,1809}$ = 0.30; *p* = .83	$F_{3,1428}$ = 0.37; *p* = .78	$F_{3,3060}$ = 0.49; *p* = .69

AUC = area under the receiver operating characteristic curve.

### Condom use and multiple sexual partners

The following effects were selected for the model of condom use at last sex: allocation arm, sexuality group, ethnicity, education level, condom use at last sexual encounter at baseline and condom use during first sex with last new partner. The interaction between allocation arm and condom use during first sex with last new partner is included (Table 3).

**Table 3.** Results of prognostic models for condom use at last sexual encounter at 1 year developed using multiply imputed datasets.

	Coefficient^†^ (95% CI)	*p*-value
Intercept/Reference*	−0.89 (−1.16, −0.62)	<0.001
**Safetxt allocation**	0.24 (0.08, 0.40)	0.003
**Sexuality group**		*<0.001*
MSW only	−0.21 (−0.44, 0.02)	0.070
WSM or WSMW	−0.58 (−0.78, −0.37)	<0.001
WSW only	−0.59 (−1.49, 0.30)	0.192
All other groups	−0.16 (−1.14, 0.83)	0.753
**Ethnicity**		*0.048*
Black/Black British	0.25 (0.06, 0.44)	0.009
Asian/Asian British	0.25 (−0.11, 0.62)	0.167
Mixed background	0.22 (−0.04, 0.48)	0.091
Other background	0.12 (−0.43, 0.67)	0.672
**Education level**		*0.001*
17 or over	0.09 (−0.10, 0.29)	0.337
Still in full time education	0.30 (0.10, 0.49)	0.003
**Condom use during last sexual encounter**	0.38 (0.21, 0.55)	<0.001
**Condom use during first sexual encounter with last new partner**	0.51 (0.31, 0.71)	<0.001
**Safetxt-condom use during first sexual encounter with last new partner interaction**	−0.24 (−0.50, 0.01)	0.058

All predictor variables are measured at baseline. Joint *p*-values are in *italics*.

CI: confidence interval; IMD: index of multiple deprivation; MSW: men who have sex with women; WSM: women who have sex with men; WSMW: women who have sex with men and women; WSW: women who have sex with women; NSU: non-specific urethritis.

†Coefficients are log-odds ratios. *Reference levels are as follows: control arm, men who have sex with men or men who have sex with men and women, white, education level of 16 years or under, at baseline used condom at last sex and used condom during first sex with last new partner.

WSW are estimated to have lowest and MSM or MSMW highest odds of condom use at last sex at 1 year. WSW, on average, have odds 0.55 times those for a MSM or MSMW (95% CI: 0.23 to 1.35, *p* = .192). Asian/Asian British participants, on average, have highest odds of condom use at last sex at 1 year: their expected odds are 1.29 times those of a white participant (95% CI: 0.90 to 1.85, *p* = .167). Black/Black British participants have 1.29 times the odds of condom use at last sex at 1 year compared to white participants (95% CI: 1.07 to 1.55, *p* = .009). Being in full time education is associated with a 1.35-fold increase in odds of condom use compared to having education of 16 years or under (95% CI: 1.11 to 1.64, *p* = .003).

The following effects were selected for the model of number of sexual partners: age, sexuality group, education level, IMD, condom use at last sexual encounter at baseline and number of sexual partners at baseline. The selected interactions were between: condom use at last sex and number of partners at baseline, age and sexuality group, and age and IMD. The interaction involving number of partners was omitted at the level of no partners at baseline due to insufficient participants with these characteristics (Table 4).

**Table 4.** Results of prognostic models for number of sexual partners at 1 year developed using multiply imputed datasets.

	Coefficient^†^ (95% CI)	*p*-value
Intercept/Reference*	−0.01 (−0.76, 0.73)	0.978
**Age (years)**	0.05 (−0.08, 0.18)	0.451
**Sexuality group**		*0.002*
MSW only	1.1 (−0.69, 0.72)	0.968
WSM or WSMW	−0.63 (−1.27, 0.01)	0.052
WSW only	−1.82 (−3.64, 0.01)	0.051
All other groups	−0.35 (−3.33, 2.64)	0.820
**Age (years)-Sexuality group interaction**		*0.057*
MSW only	−0.17 (−0.29, −0.04)	0.011
WSM or WSMW	−0.12 (−0.24, −0.01)	0.041
WSW only	0.16 (−0.20, 0.53)	0.377
All other groups	0.10 (−0.60, 0.80)	0.784
**IMD**		*0.005*
2	0.09 (−0.39, 0.58)	0.699
3	−0.47 (−0.94, 0.00)	0.050
4	−0.36 (−0.82, 0.10)	0.124
5 – most deprived	−0.63 (−1.09, −0.17)	0.007
**Age (years)-IMD interaction**		*0.012*
2	−0.01 (−0.11, 0.09)	0.843
3	0.10 (0.00, 0.20)	0.047
4	0.06 (−0.04, 0.15)	0.240
5 – most deprived	0.12 (0.03, 0.22)	0.009
**Education level**		*0.013*
17 or over	0.06 (−0.12, 0.24)	0.516
still in full time education	0.23 (0.04, 0.42)	0.016
**Condom use during last sexual encounter**	−0.48 (−0.90, −0.06)	0.024
**Number of sexual partners**		*<0.001*
0	0.18 (−1.62, 1.98)	0.845
2 or more	1.08 (0.89, 1.26)	<0.001
**Condom use during last sexual encounter-Number of sexual partners interaction**		
0	*omitted*	*omitted*
2 or more	0.50 (0.07, 0.94)	*0.024*

All predictor variables are measured at baseline. Joint *p*-values are in *italics*.

CI: confidence interval; IMD: index of multiple deprivation; MSW: men who have sex with women; WSM: women who have sex with men; WSMW: women who have sex with men and women; WSW: women who have sex with women; NSU: non-specific urethritis.

†Coefficients are log-odds ratios. *Reference levels are as follows: 16 years old, men who have sex with men or men who have sex with men and women, IMD of 1 (least deprived), education level of 16 years or under, used condom at last sex at baseline and one sexual partner at baseline.

Interaction between condom use during last sexual encounter and number of sexual partners omitted at the level of 0 partners due to a insufficient participants with these characteristics.

WSW had the lowest and MSW had the highest odds of having multiple sexual partners for 16 year olds. For every year older a participant is, their odds of having multiple partners differs by their sexuality and IMD: for the least deprived, the odds increase most with age for WSW (OR = 1.18, 95% CI: 0.82 to 1.70, *p* = .377) and decrease most with age for MSW (OR = 0.85, 95% CI: 0.75 to 0.96, *p* = .011). The odds of having multiple partners increases with age, regardless of IMD, for MSM or MSMW, WSW and all other sexuality groups. Participants still in full time education have 1.26 times the odds of having multiple partners compared to participants with education of 16 years or under (95% CI: 1.04 to 1.52, *p* = .016). Having two or more sexual partners compared to one at baseline is associated with a 2.94-fold or 4.85-fold increase in odds of having two or more partners at 1 year for those that did not (95% CI: 2.44 to 3.54, *p* < .001) and did (95% CI: 3.22 to 7.30, *p* < .001) use a condom at last sex, respectively.

Both models had fair accuracy but poor AUC and CV AUC (Table 2). There was no evidence of poor fit or calibration for either model.

## Discussion

As the risk of adverse health effects increases with number of STIs, it is useful for clinicians to know which factors are associated with higher rates of reinfection, and to what extent.^4,10^ The prognostic model developed for reinfection at 1 year identified allocation arm, age, sexuality group, being of Asian/Asian British ethnicity (for those in control group) and mixed or ‘other’ ethnicity (for those in safetxt group) or Black/Black British ethnicity (in either group), having a diagnosis of both gonorrhoea and chlamydia, IMD, whether the participant tested before sex with last new partner and whether their partner tested before sex with last new partner as significant risk factors of reinfection. The effect of age on reinfection depended on whether the participant’s most recent new partner had tested before sex with their last new partner. MSM or MSMW and those with gonorrhoea and chlamydia diagnoses at baseline had the highest odds of reinfection.

Previous studies which have developed prognostic models for STI diagnosis have tended to identify similar risk factors and have similar AUCs.^19,20^ Our study provides more recent evidence and lends support to these findings due to the size of the sample, the prospective nature of the data and having a sample with good representation across social and ethnic groups. Previous studies have found people from black and white ethnic groups to be at highest risk of STI. In contrast, our analysis revealed Asian/Asian British and mixed ethnic minorities were most likely to become re-infected for those in the control and safetxt groups, respectively. Despite having higher odds of condom use, Black/Black British participants had significantly higher odds of reinfection compared to white participants, irrespective of treatment arm and all other controlled variables (Table 1). These findings are likely to be influenced by other factors not measured in this research, for example ethnicity being a marker for social determinants of health related to medical mistrust, institutional racism, inequalities in the access to care experienced and levels of STI within sexual network.^21^ Barriers to access to healthcare for ethnic minorities can include differences in language and culture, and poor understanding within healthcare services of population diversity.^22^

Sexual behaviours such as not using condoms consistently and having multiple sexual partners are known to be associated with higher risk of STIs and other sexual health concerns.^1^ Understanding the factors associated with the differences in behaviour can in turn aid future work to target those most in need of support. A participant’s sexuality, education level and condom use at last sex at baseline were significant risk factors for these behaviours.

## Strengths and limitations

Data were from a large, representative sample of population diagnosed with chlamydia and/or gonorrhoea.^11^ The follow-up was high and any missing data were MI appropriately (Suppl 2). Risk factors that have not previously been analysed in prediction models for reinfection, condom use or number of sexual partners such as whether a participant tested before sex with last new partner and whether their partner tested before sex with last new partner were assessed. Both these testing behaviours were identified as significant predictors of reinfection.

This study contributes to the literature for modelling reinfection which is sparse compared to that for infection. Each of the existing reinfection modelling studies has weaknesses that this study improves upon: short-term follow-up,^23^ not focused on the age most at risk,^24,25^ only men,^26–28^ only women,^5,29,30^ only chlamydia,^29,31^ only gonorrhoea,^32^ only heterosexual participants.^31^ Restrictive inclusion criteria in the existing studies limits which comparisons can be made, for example between sexuality groups. Other studies have used health behaviour models to identify predictors of sexual behaviours.^33,34^ However, these were developed using expert opinion rather than a pre-specified statistical strategy.

Although the sample size of our study is large, the generalisability of the results may be limited. Our study included young individuals in the UK who presented to sexual health clinics. These are part of the free National Health Service (NHS) in the UK. There may be systematic differences between individuals who present to these clinics and the general population. We suggest that further studies should be conducted to improve understanding for other age groups and for populations outside of the UK.

All the models’ calibrations and fits were very good, but the discrimination was relatively weak (AUCs between 0.60 and 0.70). All models were internally (but not externally) validated.

Identification of the risk factors in this paper can be used to control for confounding in future mediation analyses to assess the extent of safetxt’s indirect effect through sexually risky behaviours on reinfection.

## Conclusions

This paper contributes to the understanding of risk factors for STIs and sexual behaviours in young people. Demographic data such as age, ethnicity and IMD were associated with risk of reinfection. Having both gonorrhoea and chlamydia diagnoses at baseline, testing behaviours of the participant and their partner, and a participant’s sexuality were also identified as key predictors of differences in risk of reinfection. Two sexual behaviours were analysed: condom use at last sex and having multiple sexual partners. Risk factors of these included sexuality, education level and condom use at last sexual encounter at baseline. Greater understanding of the factors associated with STI reinfections and sexual behaviours can aid the development of interventions.

## Supplemental Material

**Supplemental Material** - Factors associated with sexually transmitted reinfections, number of sexual partners and condom use among previously infected young people Supplemental Material for Factors associated with sexually transmitted reinfections, number of sexual partners and condom use among previously infected young people by Isobel Landray, James R. Carpenter, Sima Berendes, Melissa J. Palmer and Caroline Free in International Journal of STD & AIDS

**Supplemental Material** - Factors associated with sexually transmitted reinfections, number of sexual partners and condom use among previously infected young people Supplemental Material for Factors associated with sexually transmitted reinfections, number of sexual partners and condom use among previously infected young people by Isobel Landray, James R. Carpenter, Sima Berendes, Melissa J. Palmer and Caroline Free in International Journal of STD & AIDS

**Supplemental Material** - Factors associated with sexually transmitted reinfections, number of sexual partners and condom use among previously infected young people Supplemental Material for Factors associated with sexually transmitted reinfections, number of sexual partners and condom use among previously infected young people by Isobel Landray, James R. Carpenter, Sima Berendes, Melissa J. Palmer and Caroline Free in International Journal of STD & AIDS

**Supplemental Material** - Factors associated with sexually transmitted reinfections, number of sexual partners and condom use among previously infected young people Supplemental Material for Factors associated with sexually transmitted reinfections, number of sexual partners and condom use among previously infected young people by Isobel Landray, James R. Carpenter, Sima Berendes, Melissa J. Palmer and Caroline Free in International Journal of STD & AIDS

**Supplemental Material** - Factors associated with sexually transmitted reinfections, number of sexual partners and condom use among previously infected young people Supplemental Material for Factors associated with sexually transmitted reinfections, number of sexual partners and condom use among previously infected young people by Isobel Landray, James R. Carpenter, Sima Berendes, Melissa J. Palmer and Caroline Free in International Journal of STD & AIDS

## Data Availability

Individual deidentified patient data, including a data dictionary, will be made available via our data sharing portal FreeBIRD website indefinitely. The trial protocol, statistical analysis plan, and trial publications will be available online. The Stata code for the analyses in this manuscript will be made available upon reasonable request.*
