# Supplementary material for: Factors associated with sexually transmitted reinfections, number of sexual partners and condom use among previously infected young people
Source: Int J STD AIDS. 2025 Jun 11;36(10):808–15. doi: 10.1177/09564624251348693 (PMC12374008; doi:10.1177/09564624251348693)
Supplement: Supplemental Material - Factors associated with sexually transmitted reinfections, number of sexual partners and condom use among previously infected young people [file sj-pdf-1-std-10.1177_09564624251348693.pdf]

Figure S1: Variables recorded in safetxt trial data, Free et al. (2022) [1]

Timepoint	Variable type	Variables recorded
**Baseline**	***Allocation***	Intervention/control arm
	Demographic	Age, sexuality*, gender, ethnicity, type of infection, education level, index of multiple deprivation.
	Sexual health	Condom use during last sexual encounter, condom use during first sexual encounter with last new partner, tested before sex with last new partner, number of partners in past 12 months.
	Contact details	Provision of: email address, alternative email address, alternative mobile number.
**Across the series of text messages†**	***Knowledge related to STIs***	If someone had an STI, they would know; STIs are rare; I can tell if someone has an STI
	Attitude toward partner notification	Most people with STI will tell their partner; It is my responsibility to tell my partner if I had an STI; My partner would be glad I let them know I had an STI; My partner would think badly of me
	Self-efficacy in telling partner about an infection	Ease to tell last partner you had STI; Ease to tell partner to get treatment; Ease to tell new partner you had STI; Ease to tell new partner to get treatment
	Correct condom use self-efficacy	Ease to put condom on; Ease to stop condom drying out; Ease to stop condom breaking/coming off; Ease to keep condom on while withdrawing; Ease to keep condom on start to finish
	Self-efficacy in negotiating condom use	Ease telling partner you want to use condoms; Ease telling new partner you want to use condoms; Ease telling new partner you won't have sex unless use condoms
	Reading intervention content	Did anyone else read the messages we sent you?; How did you feel about them reading the messages?; Did you know anyone else who took part in the study?; Did they read the messages we sent you?; Did you read the messages we sent you?; How many of the messages did you read?
**4 weeks follow-up**	***Secondary outcomes***	Correctly treated for STI; participant told last partner they had sex with before testing positive to get treatment; partner attended clinic for treatment; condom use at last sexual encounter
	Other	Did you take the treatment for STI?; Did you avoid sex for 7 days after treatment?; Was a condom used last time you had sex?; Number of sexual partners since joining the study
**1 year follow-up**	***Primary outcome***	Cumulative incidence of chlamydia or gonorrhoea reinfection
	Secondary outcomes	Condom use at last sexual encounter; ≥2 sexual partners since joining the trial; sex with someone new since joining the trial; condom use at first sex

		with most recent partner; STI testing for self, before first sexual encounter with most recent new partner (self-reported and testing confirmed by clinic record); most recent new partner was tested for STI before sex with participant; road traffic accident in past year when participant was driver; experience of partner violence in past year; diagnosis of "any" STI after joining trial according to postal test results and clinic records

†Each variable at this timepoint is separated by ';'. The variables measured are statements or questions which participants answered or rated on appropriate scales from 1 to 5 e.g. from strongly disagree to strongly agree. These question items were then summed to give a score for each variable group.

* Sexuality was grouped into the following: MSM or men-who-have-sex-with-men-and-women (MSMW); men-who-have-sex-with-women-only (MSW); women-who-have-sex-with-men (WSM) or women-who-have-sex-with-men-and-women (WSMW); women-who-have-sex-with-women-only (WSW); all other groups (non-binary individuals and individuals that did not state their sexuality were grouped due to data sparsity).

1. Free C, Palmer M J, McCarthy O L, et al. Effectiveness of a behavioural intervention delivered by text messages (safetxt) on sexually transmitted reinfection in people aged 16-24 years: randomised controlled trial. BMJ. 2022;378.
