# Supplementary material for: Factors associated with sexually transmitted reinfections, number of sexual partners and condom use among previously infected young people
Source: Int J STD AIDS. 2025 Jun 11;36(10):808–15. doi: 10.1177/09564624251348693 (PMC12374008; doi:10.1177/09564624251348693)
Supplement: Supplemental Material - Factors associated with sexually transmitted reinfections, number of sexual partners and condom use among previously infected young people [file sj-pdf-2-std-10.1177_09564624251348693.pdf]

<u>Choice of imputation model</u>

1. <u>Introduction</u>

The outcome as well as any of the potential predictors with missing values for each model are multiply imputed. The binary variables are imputed using logistic regression. The ordinal variables are treated as continuous as convergence issues are expected with ordinal logistic regression. Therefore, all non-binary variables with missing data are imputed using linear regression. For categorical variables, the imputed values are rounded.

The imputation models are fitted separately by allocation arm and age category with all the other potential predictors as covariates. Fitting the imputation models by allocation arm and age category which are fully observed improves the compatibility of the imputation model with the substantive model [1]. This is equivalent to fitting the imputation model with all interactions between allocation arm and age category with the other predictors. The variables recorded throughout the study can improve the MICE if associated with the variables being imputed and if observed when the other variables are missing (Suppl. Figure 1) [1]. A backwards stepwise selection was implemented to select additional useful covariates for the imputation model from the data: only variables that tended to be observed when the outcome of interest was missing were considered for inclusion in the imputation models. (Suppl. 2 details the choice of imputation models.)

In this analysis, the 100 imputed datasets are ‘stacked’ and the model selection process detailed in Section 2.4.3 is used on the whole dataset. The estimators are asymptotically equivalent to the MI estimators [2] so variable selection is legitimate based on the coefficients estimated from the stacked data [1]. For the tests used in model selection, the standard errors are adjusted by weighting each observation by 0.01 (the inverse of the number of imputed datasets). Assuming relatively small amounts of missingness, this is a valid way in which to account for the fact that the sample size is only one hundredth the size of the stacked dataset [3].

To impute missing data prior to prognostic model development, all the potential predictors considered for the substantive model are included in the imputation model. In addition, including variables that are predictive of the data being missing and predictive of the actual variables with missing data can improve the imputation model.

This appendix summarises the variables selected for each imputation model. In each case, the focus is on selecting variables predictive of the actual value of the outcome of the substantive

model. It is also important these variables are observed when the variable with missing data is not.

2. Prognostic model for reinfection at 1 year:

Method: For the reinfection model, the imputation model used in the main analysis is first considered (i.e. with the score for 'attitude' and the number of sexual partners at 4 weeks as auxiliary variables). Whether a participant tested at baseline was also included as an auxiliary variable in the imputation model in the main analysis, but in this prognostic modeling it is already included as it is in the substantive model. Then, as education level is a potential predictor and has missing data, predictors of this variable's values and whether it is missing are investigated to be added to the imputation model.

Results: From the primary trial analysis, we know none of the 'contact details' variables from baseline were identified as important predictors of reinfection at 1 year. Of the intermediate variables, the number of sexual partners at 4 weeks and the 'attitude' score were identified as important predictors of reinfection at 1 year. For 70.8% of participants with missing reinfection data, their number of sexual partners at 4 weeks is recorded. For 70.2% of participants with missing reinfection data, their 'attitude' is recorded. The 'knowledge' score was added to the imputation model as it was identified as a key predictor of education level.

3. Prognostic models for condom use at last sex and number of sexual partners at 1 year:

Method: For the condom use and number of partners models, backwards stepwise selection is implemented twice. Firstly, with the model (logistic in all cases in this project) with the outcome of the substantive model as the outcome, the potential predictors to be investigated 'locked in' and the 'contact details' variables from baseline (Figure S1). Then, any selected 'contact details' are additionally 'locked in' before conducting the second round of backwards stepwise selection with variables measured at 4 weeks. This choice is made as in the primary trial analysis as when one variable was not recorded at 1 year, almost all other 1 year variables were also missing. The difference between this imputation model development and that of the primary trial analysis is that not all baseline variables were included in their substantive models so the first selection process included those other variables. Terms with $p>0.05$ are eligible for removal from the models in the backwards stepwise selections.

Results:

Condom use at last sex: None of the 'contact details' variables from baseline were identified as important predictors of condom use at last sex at 1 year. Of the 4 week variables, the condom use

at last sex at 4 weeks and whether the participant completed the correct treatment were identified as important predictors of condom use at last sex at 1 year. For 79.9% of participants with missing condom use at last sex data at 1 year, their condom use at last sex at 4 weeks is recorded. For 79.9% of participants with missing condom use at last sex data at 1 year, whether they completed the correct treatment is recorded. Therefore, both are 'helpful' variables as they are observed some of the time when the outcome is not. Inclusion of both variables may not improve the imputation model further than the inclusion of just one of these variables. For participants with missing condom use at last sex at 1 year, when one of the additional variables identified (condom use at last sex at 4 weeks or correct treatment) is missing, approximately 99% of participants also have the other missing.

Number of sexual partners: None of the 'contact details' or 4 week variables from baseline were identified as important predictors of number of sexual partners at 1 year. As no additional variables were selected from the usual list, the other 1 year variables were tabulated against the number of sexual partners at 1 year. The 1 year variable that was most observed when the number of sexual partners at 1 year was missing was whether the participant had any STI diagnosis. Therefore, a third backwards stepwise selection was conducted and this variable was added to the imputation model. For 19.2% of participants with missing number of sexual partners data at 1 year, whether they had any STI diagnosis at 1 year is recorded. Therefore, this variable is fairly 'helpful' as it is predictive of the substantive model outcome and observed some of the time when the outcome is not.

4. Strengths and limitations of imputation models

The imputation models were only fitted by age and allocation which may have limited the number of interactions selected [1]. Interactions between other variables are less likely to be selected in the variable selection process as they will not have been imputed compatibly which could lead to bias in the variables selected. Although this is clearly a limitation, in this context the most plausible and interesting interactions involve allocation and/or age [4, 5]. Further, it is better to be cautious in the inclusion of interactions due to multiple testing and model instability [6].

Substantive model compatible fully conditional specification (SMCFCS) would have been a more thorough approach to imputation [7]. Due to small subgroups in some levels of the predictors, SMCFCS's computational intensity meant MICE by allocation and age category was favoured [7].

Multiple imputation counters the issues that using only complete records can result in: reduction in the power which can lead to failure to detect important predictors and biased estimates. The multiple imputation analyses also make a weaker assumption about the missing data than if only complete records were used: data are assumed missing at random, rather than missing completely at random.

References

1. Carpenter J R, Bartlett J, Morris T, et al. Multiple Imputation and Its Application. Second ed. Hoboken, New Jersey: John Wiley Sons, Inc.; 2023.
2. Wang N, Robins J M. Large-sample theory for parametric multiple imputation procedures. Biometrika. 1998;85(4):935-48.
3. Wood A M, White I R, Royston P. How should variable selection be performed with multiply imputed data? Statistics in Medicine. 2008;27(17):3227-48.
4. Harrell F E. Regression modelling strategies. Second ed: Springer International Publishing; 2015.
5. Shipe M E, Deppen S A, Farjah F, et al. Developing prediction models for clinical use using logistic regression: an overview. Journal of thoracic disease. 2019;11:S574-S84.
6. Riley R D, Collins G S. Stability of clinical prediction models developed using statistical or machine learning methods. Biometrical Journal. 2023;65(8).
7. Bartlett J W, Seaman S R, White I R, et al. Multiple imputation of covariates by fully conditional specification: accommodating the substantive model. Statistical Methods in Medical Research. 2015;24(4):462-87.
