# Supplementary material for: Factors associated with sexually transmitted reinfections, number of sexual partners and condom use among previously infected young people
Source: Int J STD AIDS. 2025 Jun 11;36(10):808–15. doi: 10.1177/09564624251348693 (PMC12374008; doi:10.1177/09564624251348693)
Supplement: Supplemental Material - Factors associated with sexually transmitted reinfections, number of sexual partners and condom use among previously infected young people [file sj-pdf-3-std-10.1177_09564624251348693.pdf]

Model assessment measures

Model assessment measures can be used to evaluate the prediction models developed. However, the usual measures need to be implemented and calculated differently when working with multiply imputed datasets. Multiple imputation (MI) estimates of model assessment measures were obtained by Rubin's rules [1].

Accuracy: The accuracy is the simplest assessment measure and is the proportion of individuals' predicted outcomes which agree with their observed outcomes.

Calibration: The goodness of fit is tested using Hosmer-Lemeshow's (HL) test [2]. The HL test compares a test statistic to the chi-squared distribution. To combine chi-squared statistics over multiple imputed datasets into a statistic which is compared to an F-distribution to calculate a p-value, formulae originally proposed by Li et al. were utilised [3]-[5].

Discrimination: The receiver operating characteristic (ROC) curve demonstrates how a balance of sensitivity and specificity can be achieved as the classification rule moves from classifying all participants as having the outcome to all as not. The area under the ROC curve (AUC) is reported to summarise the model's predictive ability. When the AUC is closer to 1 this is indicative of very good discrimination. The AUC estimates from each imputed dataset are averaged on the log-odds scale and then back-transformed to avoid any boundary-related issues.

Cross-validation: AUC was additionally calculated using cross-validation (CV) with ten folds. Multiple imputation is performed separately in the development and validation datasets each time one of the ten folds is left out to ensure the imputation of missing data in each set is not influenced by data in the other set [1]. The prognostic model is fitted ten times with each imputed fold being held out once at a time. The AUC is calculated for the corresponding imputed fold that was left out. The multiple imputation is conducted ten times so there are 100 estimates of the AUC overall which can be averaged according to Rubin's rules [6]. Only ten rather than 100 imputations are used due to the computational cost of imputing data. If convergence issues are met due to folds being a tenth of the size of those in the original analysis, then those variables not selected for the final prognostic model are excluded from the imputation model and models will only be fit by allocation rather than by allocation and age category in CV.

Both good discrimination and calibration are required for a prognostic model to be useful. A model needs to be able to distinguish which of two participants is at greater risk *and* estimate

the risk itself well.

References

1. Carpenter J R, Bartlett J, Morris T, et al. Multiple Imputation and Its Application. Second ed. Hoboken, New Jersey: John Wiley Sons, Inc.; 2023.
2. Hosmer D W, Hosmer T, Le Cessie S, et al. A comparison of goodness-of-fit tests for the logistic regression model. Statistics in Medicine. 1997;16(9):965-980. doi:10.1002/(sici)1097-0258(19970515)16:9(965::aid-sim509)3.0.co;2-o.
3. Li K-H, Meng X-L, Raghunathan T, et al. Significance levels from repeated p-values with multiply imputed data. Statistica Sinica. 2009;1:65-92.
4. Marshall A, Altman D, Holder R, et al. Combining estimates of interest in prognostic modelling studies after multiple imputation: current practice and guidelines. BMC Medical Research Methodology. 2009;9(57). doi:10.1186/1471-2288-9-57.
5. Ratitch B, Lipkovich I, O'Kelly M. Combining analysis results from multiply imputed categorical data. PharmaSUG. 2013; paper SP03.
6. Rubin D. Multiple imputation after 18+ years. Journal of American Statistical Association. 1996;91(434):473-489. doi: 10.2307/2291635.
