# Supplementary material for: Factors associated with sexually transmitted reinfections, number of sexual partners and condom use among previously infected young people
Source: Int J STD AIDS. 2025 Jun 11;36(10):808–15. doi: 10.1177/09564624251348693 (PMC12374008; doi:10.1177/09564624251348693)
Supplement: Supplemental Material - Factors associated with sexually transmitted reinfections, number of sexual partners and condom use among previously infected young people [file sj-pdf-4-std-10.1177_09564624251348693.pdf]

Table S1: Table of baseline characteristics reproduced from Table 1 of Free et al. (2022) [1]

	Intervention (n=3123)	Control (n=3125)
**Age group** (years)		
16-19	1189 (38.1)	1117 (35.7)
20-24	1934 (61.9)	2008 (64.3)
**Mean (SD) age (years) (based on integer)**	20.3 (2.1)	20.4 (2.1)
**Gender**		
Female	2047 (65.5)	2020 (64.6)
Male	1065 (34.1)	1097 (35.1)
Non-binary	11 (0.4)	8 (0.3)
**Ethnicity**		
White British/ Other White	2428 (77.7)	2436 (78.0)
Black/Black British – Caribbean, African, other	380 (12.2)	347 (11.1)
Asian/Asian British – Bangladeshi, Chinese, Indian, Pakastani, other	89 (2.8)	91 (2.9)
Mixed	174 (5.6)	205 (6.6)
Other	52 (1.7)	46 (1.5)
**Index of multiple deprivation fifth***	n=3099	n=3096
1st (least deprived)	439 (14.2)	424 (13.7)
2nd	516 (16.7)	527 (17.0)
3rd	608 (19.6)	590 (19.1)
4th	768 (24.8)	761 (24.6)
5th (most deprived)	768 (24.8)	794 (25.6)
**Educational level†**	n=2996	n=2990
Primary and secondary (age ≤ 16 years)	436 (14.6)	450 (15.1)
Secondary onwards (age ≥ 17 years)	1352 (45.1)	1348 (45.1)
Still in full time education	1208 (40.3)	1192 (39.9)
**Gender and sexual orientation**		
Women who have sex with men only	1901 (60.9)	1855 (59.4)
Men who have sex with women only	790 (25.3)	778 (24.9)

Women who have sex with women only	20 (0.6)	17 (0.5)
Men who have sex with men only	226 (7.2)	258 (8.3)
Women who have sex with women and men	125 (4.0)	147 (4.7)
Men who have sex with women and men	49 (1.6)	60 (1.9)
Those with non-binary gender who have sex with men	7 (0.2)	3 (0.1)
Those with non-binary gender who have sex with women	1 (0)	2 (0.1)
Those with non-binary gender who have sex with women and men	3 (0.1)	3 (0.1)
Not stated	1 (0)	2 (0.1)
**Baseline diagnosis**		
Chlamydia	2449 (78.4)	2433 (77.9)
Gonorrhoea	283 (9.1)	303 (9.7)
Gonorrhoea and chlamydia	159 (5.1)	155 (5.0)
Gonorrhoea or non-specific urethritis	27 (0.9)	32 (1.0)
Non-specific urethritis	125 (4.0)	123 (3.9)
Unknown	80 (2.6)	79 (2.5)
**Condom used during last sexual encounter**		
Yes	747 (23.9)	806 (25.8)
No	2314 (74.1)	2273 (72.7)
Unsure	62 (2.0)	46 (1.5)
**Condom used during first sexual encounter with last new partner**		
Yes	981 (31.4)	1035 (33.1)
No	2065 (66.1)	2010 (64.3)
Unsure	77 (2.5)	80 (2.6)
**Tested before sex with last new partner**		
Yes	1242 (39.8)	1243 (39.8)
No	1798 (57.6)	1787 (57.2)
Unsure	83 (2.7)	95 (3)
**Partner tested before sex last new partner**	n=3120	n=3125

Yes	437 (14)	457 (14.6)
No	1189 (38.1)	1181 (37.8)
Unsure	1494 (47.9)	1487 (47.6)
**No of partners in past 12 months**	n=3120	n=3122
0	5 (0.2)	2 (0.1)
1	496 (15.9)	538 (17.2)
≥2	2619 (83.9)	2582 (82.7)

Values are reported as n (%) unless otherwise stated. SD = standard deviation.

*Reduced denominator – Index of multiple deprivation fifth was missing for some participants who provided an invalid postcode.

†Reduced denominator – education information was missing for some participants due to non-response.

1. Free C, Palmer M J, McCarthy O L, et al. Effectiveness of a behavioural intervention delivered by text messages (safetxt) on sexually transmitted reinfection in people aged 16-24 years: randomised controlled trial. BMJ. 2022;378.
