# Supplementary material for: Factors associated with sexually transmitted reinfections, number of sexual partners and condom use among previously infected young people
Source: Int J STD AIDS. 2025 Jun 11;36(10):808–15. doi: 10.1177/09564624251348693 (PMC12374008; doi:10.1177/09564624251348693)
Supplement: Supplemental Material - Factors associated with sexually transmitted reinfections, number of sexual partners and condom use among previously infected young people [file sj-pdf-5-std-10.1177_09564624251348693.pdf]

Table S2: Table of subset of primary and secondary outcomes from safetxt trial, reproduced from Table 2 of Free et al. (2022) [1]

Outcomes	Safetxt group (n=3123)	Control group (n=3125)	Odds ratio (95% CI)	P value
**Primary outcome (1 year)**				
Cumulative incidence of chlamydia or gonorrhoea reinfection	693 (22.2)	633 (20.3)	1.13 (0.98 to 1.31)	0.09
**Secondary outcomes (1 year)**				
Condom use at last sexual encounter	1056 (33.8)	975 (31.2)	1.14 (1.01 to 1.28)	0.04
≥2 sexual partners since joining the trial	1777 (56.9)	1713 (54.8)	1.11 (1.00 to 1.24)	0.06

Note: Results are only presented for the outcomes for which prediction models are developed in this paper.

Values are n (%) estimated from imputed data. Analyses are based on intention to treat principle. Odds ratios are adjusted for pre-specified baseline covariates (age, type of STI at baseline, sexual orientation and ethnicity).

CI = confidence interval.

1. Free C, Palmer M J, McCarthy O L, et al. Effectiveness of a behavioural intervention delivered by text messages (safetxt) on sexually transmitted reinfection in people aged 16-24 years: randomised controlled trial. BMJ. 2022;378.
